# Disrupted Ca^2+^ homeostasis and immunodeficiency in patients with functional IP_3_ receptor subtype 3 defects

**DOI:** 10.1038/s41423-022-00928-4

**Published:** 2022-10-27

**Authors:** Julika Neumann, Erika Van Nieuwenhove, Lara E. Terry, Frederik Staels, Taylor R. Knebel, Kirsten Welkenhuyzen, Kourosh Ahmadzadeh, Mariah R. Baker, Margaux Gerbaux, Mathijs Willemsen, John S. Barber, Irina I. Serysheva, Liesbeth De Waele, François Vermeulen, Susan Schlenner, Isabelle Meyts, David I. Yule, Geert Bultynck, Rik Schrijvers, Stephanie Humblet-Baron, Adrian Liston

**Affiliations:** 1 VIB Center for Brain and Disease Research, Leuven, Belgium; 2 Department of Microbiology and Immunology, KU Leuven, Leuven, Belgium; 3 UZ Leuven, Leuven, Belgium; 4 Department of Pharmacology and Physiology, University of Rochester, Rochester, NY 14526 USA; 5 Laboratory of Molecular and Cellular Signaling, Department of Cellular and Molecular Medicine, Leuven Kankerinstituut, KU Leuven, Leuven, Belgium; 6 Laboratory of Immunobiology, Department Microbiology and Immunology, Rega Institute, KU Leuven, Leuven, Belgium; 7 Department of Biochemistry and Molecular Biology, Structural Biology Imaging Center, McGovern Medical School at The University of Texas Health Science Center at Houston, Houston, TX 77030 USA; 8 Pediatric Department, Academic Children Hospital Queen Fabiola, Université Libre de Bruxelles, Brussels, Belgium; 9 Department of Pediatric Neurology, University Hospitals Leuven, Leuven, Belgium; 10 Department of Pulmonology, University Hospitals Leuven, Leuven, Belgium; 11 Laboratory for Inborn Errors of Immunity, Department of Immunology and Microbiology, KU Leuven, Leuven, Belgium; 12 Laboratory for Allergy and Clinical Immunology and Immunogenetics Research Group, Department of Microbiology, Immunology and Transplantation, KU Leuven, Leuven, Belgium; 13 Immunology Programme, The Babraham Institute, Babraham Research Campus, Cambridge, CB22 3AT UK

**Keywords:** Primary immunodeficiency, Calcium signalling, Whole exome sequencing, Adaptive immunity, Primary immunodeficiency disorders, Calcium signalling, Immunogenetics

## Abstract

Calcium signaling is essential for lymphocyte activation, with genetic disruptions of store-operated calcium (Ca^2+^) entry resulting in severe immunodeficiency. The inositol 1,4,5-trisphosphate receptor (IP_3_R), a homo- or heterotetramer of the IP_3_R1-3 isoforms, amplifies lymphocyte signaling by releasing Ca^2+^ from endoplasmic reticulum stores following antigen stimulation. Although knockout of all IP_3_R isoforms in mice causes immunodeficiency, the seeming redundancy of the isoforms is thought to explain the absence of variants in human immunodeficiency. In this study, we identified compound heterozygous variants of *ITPR3* (a gene encoding IP_3_R subtype 3) in two unrelated Caucasian patients presenting with immunodeficiency. To determine whether *ITPR3* variants act in a nonredundant manner and disrupt human immune responses, we characterized the Ca^2+^ signaling capacity, the lymphocyte response, and the clinical phenotype of these patients. We observed disrupted Ca^2+^ signaling in patient-derived fibroblasts and immune cells, with abnormal proliferation and activation responses following T-cell receptor stimulation. Reconstitution of IP_3_R3 in IP_3_R knockout cell lines led to the identification of variants as functional hypomorphs that showed reduced ability to discriminate between homeostatic and induced states, validating a genotype–phenotype link. These results demonstrate a functional link between defective endoplasmic reticulum Ca^2+^ channels and immunodeficiency and identify IP_3_Rs as diagnostic targets for patients with specific inborn errors of immunity. These results also extend the known cause of Ca^2+^-associated immunodeficiency from store-operated entry to impaired Ca^2+^ mobilization from the endoplasmic reticulum, revealing a broad sensitivity of lymphocytes to genetic defects in Ca^2+^ signaling.

## Introduction

Genetic studies of patients with severe immunodeficiency have led to the identification of defects in Ca^2+^ signaling as key causes of T-cell and B-cell functional deficiency [1, 2]. Ca^2+^ signaling is not a single event but the result of orchestrated spatiotemporal changes in Ca^2+^ flux from different cellular and extracellular compartments [3] acting in concert with other signaling pathways [4]. In lymphocytes, the key event following the engagement of a T-cell receptor (TCR) or B-cell receptor (BCR) is the elevation of the cytosolic Ca^2+^ concentration ([Ca^2+^]_cyt_) [5, 6]. Identifying the genes involved in T-cell and B-cell immunodeficiency sheds light on the rate-limiting biochemical steps critical for this activation process.

The Ca^2+^ flux triggered by TCR and BCR engagement is realized via both Ca^2+^release from intracellular Ca^2+^ stores and influx from the extracellular compartment in multiple phases. The first step is mediated by the second messenger inositol 1,4,5-trisphosphate (IP_3_), which is generated upon activation of phospholipase C γ [7]. IP_3_ binds to and opens tetrameric IP_3_ receptors (IP_3_Rs), thereby releasing Ca^2+^ from endoplasmic reticulum (ER) stores into the cytosol [8, 9]. Although this event only transiently increases [Ca^2+^]_cyt_, in the second stage, the ER transmembrane (TM) protein stromal interaction molecule 1 (STIM1) senses lower ER [Ca^2+^], and via a conformational change directly triggers the opening of plasmalemmal ORAI1 channels [10–12]. ORAI1 is a calcium-release activated Ca^2+^ (CRAC) channel that mediates the influx of extracellular Ca^2+^, a process known as store-operated Ca^2+^ entry. This sustained increase in [Ca^2+^]_cyt_ triggers downstream signaling, notably the NF-κB and calcineurin/nuclear factor of activated T cells (NFAT) pathways [13, 14], thereby activating antigen-stimulated lymphocytes. In principle, defects in any step in the distal pathway between TCR stimulation and NFAT nuclear translocation can result in a dysfunctional immune response. Most commonly, however, genetic drivers of immunodeficiency originate from points of the pathway with rate-limiting and nonredundant single-protein bottlenecks. Furthermore, with Ca^2+^ signaling being a critical pathway in a multitude of physiological processes, from neuron excitation to cellular apoptosis [3], only variants that confer immunodeficiency without preventing fetal development are observed.

Due to the centrality of Ca^2+^ regulation, a large diversity of genetic disorders are associated with disrupted Ca^2+^ pathways [15–17]. Genetic defects in extracellular Ca^2+^ influx have been formally associated with primary immunodeficiency, with defects in the store-operated Ca^2+^ entry stage of Ca^2+^ signaling through the demise or loss-of-function of ORAI1 or STIM1 causing severe immunodeficiency [18–20] and defective nuclear translocation of NFAT blocking the production of cytokines, resulting in immunodeficiency [21]. Patients with immunodeficiency cannot mount effective immune responses, but many of these patients also present symptoms associated with autoimmune conditions such as autoimmune cytopenia or inflammatory bowel disease [22, 23]. These outcomes may indicate the crucial role played by intact TCR signaling not only in immunogenic processes but also in tolerogenic processes such as central and peripheral tolerance [24, 25]. In contrast, primary ER Ca^2+^ release through IP_3_R channels, although associated with several nonimmunological disorders [26, 27], has yet to be linked to primary immunodeficiencies. *ITPR1*, *ITPR2* and *ITPR3* variants have been suggested to cause (spino-)cerebellar ataxia [28, 29], anhidrosis [30], and Charcot–Marie–Tooth disease [31], respectively. In mice, lymphocyte defects were not observed when individual genes were knocked out; in contrast, they were observed only in triple-knockout mice [32–34]. Based on the 60–80% sequence homology among the isoforms [35] and the formation of either homo- or heterotetramers, subunits may show redundancy in lymphocytes. However, whether lymphocytes are susceptible to genetic variants that can impair primary Ca^2+^ efflux from the ER in humans remains unknown.

In this study, we identified two unrelated patients with immunodeficiency and immune dysregulation who harbored compound heterozygous *ITPR3* variants. We found that the three identified variants differentially impact channel function and intracellular calcium homeostasis, with proliferation and activation defects observed in stimulated lymphocytes. Together, these results demonstrate that human lymphocytes are susceptible to genetic defects in the primary ER Ca^2+^ release system, with niche-filling *ITPR3* variants altering IP_3_R sensitivity. This study thus establishes genetic defects in IP_3_Rs as a new class of inborn errors of immunity.

## Materials and methods

Written informed consent was obtained from all participants, and the Ethics Committee of UZ/KU Leuven approved the study (S52653, S58466).

### Whole-exome sequencing and analysis

For the kindred of P1, whole-exome sequencing was performed using genomic DNA isolated from whole blood using the QIAmp DNA Blood Midi kit (Qiagen, Hilden, Germany) according to the manufacturer’s instructions. The sequencing library was prepared using ligation-mediated PCR and hybridized to the SureSelect Biotinylated RNA library (Agilent Technologies, Santa Clara, CA) for enrichment by BGI (Shenzhen, China) before paired-end sequencing on a HiSeq2000 (Illumina, San Diego, CA) platform. The generated FastQ files were mapped to the human genome version 19 (hg19) using the Burrows-Wheeler Aligner (BWA, v0.7.5a). Duplicates were removed using Picard MarkDuplicates, and realignment was performed according to the Genome Analysis Toolkit 3.1 best practices guidelines. Variants were called using HaplotypeCaller and coding nonsynonymous variants with a high CADD score and a mean allelic frequency of <0.005 in the gnomAD database were retained.

For the kindred of P2, whole-exome sequencing was performed by Macrogen (Seoul, Korea). Exome capture using SureSelect Human All Exon V7 (Agilent) preceded paired-end sequencing on a NovaSeq6000 (Illumina) platform. A computational pipeline was developed using bcbio-nextgen as a backend (https://github.com/bcbio/bcbio-nextgen, v1.1.5-b) to process the read data and perform tasks such as quality control, variant discovery, annotation, and filtering. Briefly, the sequencing reads in FASTQ format were aligned to the human reference genome (GRCh37) using BWA (v0.7.17). The resulting BAM files were further processed to remove duplicate reads using biobambam (v2.0.87). The resulting variants were annotated using snpEff (v4.3.1t) for function prediction and vcfanno (v0.3.1) to add information from public databases such as dbSNP (v151), dbNSFP (v3.5a), ExAC, gnomAD (v r2.1), 1000 genomes (v3), and ClinVar (v2019-05-13). Likely disease-causing variants were selected and prioritized based on quality score, allele frequency, functional impact, and probable inheritance model.

Sanger confirmation for individuals from both kindreds was performed by amplification of variant-specific gene products by PCR using KOD polymerase (Sigma‒Aldrich, St. Louis, MO, US), and the primers are listed in Supplementary Table 5. After gel purification with the NucleoSpin Gel and PCR Clean-up (Macherey-Nagel, Düren, Germany), according to the manufacturer’s instructions, sequencing was performed by Eurofins (Ebersberg, Germany).

### Variant effect prediction on protein structure

The cryo-EM structure of IP_3_R3 with the PDB accession number 6DQJ was used to analyze the identified variants. Computational mutagenesis was performed in COOT [36], and subsequent molecular refinements to R2524C were performed in PHENIX [37]. Calculations of electrostatic potential and all molecular visualizations were generated using UCSF Chimera [38].

### RNA isolation and quantification

Total RNA was isolated from PBMCs and primary fibroblast cell lines generated from skin biopsy samples using TRIzol reagent (Ambion, Thermo Fisher, Waltham, MA) according to the manufacturer’s protocol. Complementary DNA was synthesized using the GoScript^TM^ Reverse Transcription System (Promega, Madison, WI). Quantitative PCR was performed on a StepOnePlus real-time PCR system (ABI, Thermo Fisher) with Fast SYBR Green Master Mix (Applied Biosystems, Foster City, CA) supplemented with gene-specific primers (see Supplementary Table 6). Experiments were performed in duplicate and repeated three times. Gene expression was calculated using the 2^–∆∆Ct^ method and normalized to the mean of two housekeeping genes before normalizing to the mean expression of all healthy controls across experiments.

### Western blotting

Primary fibroblasts and PBMCs were solubilized in lysis buffer containing 20 mM Tris-HCl, pH 7.5; 150 mM NaCl; 1% Triton-X; 1.5 mM MgCl_2_; 0.5 mM DTT; and protease inhibitor. Protein concentrations were determined by BCA, and 20 µg of protein per sample was separated on a 3–8% Tris-acetate gel. Proteins were blotted on a PVDF membrane and incubated with protein-specific primary antibodies after blocking. The primary antibodies, used at a 1/1000 dilution, included an in-house made rabbit anti-IP_3_R1 Rbt03antibody [39]. In addition, rabbit anti-IP_3_R2 (Abicode C2) and mouse anti-IP_3_R3 (IP3R3, BD Biosciences) antibodies were obtained. A rabbit pan-IP_3_R Rbt475 antibody raised against a peptide corresponding to amino acids 127–141 of human IP_3_R1 was produced in house [40, 41]. Vinculin (Sigma) was used as a loading control at a 1/2000 dilution. Secondary goat anti-mouse/rabbit IgG (Thermo Scientific, 1/10,000 dilution) were coupled to HRP. Membranes were developed using the Pierce^TM^ ECL kit (Thermo Fisher) with a ChemiDoc MP Imaging system (Bio-Rad, Hercules, CA). Quantification of the protein expression was performed with ImageJ software [42, 43]. The fold change in protein expression was normalized using Excel to the mean of all healthy controls within each independent experiment.

HEK-3KO cells, HEK293 cells expressing only endogenous IP_3_R3, and HEK-3KO cells stably expressing IP_3_R constructs were solubilized in RIPA lysis buffer supplemented with Halt^TM^ protease inhibitor cocktail (Thermo Fisher). Protein concentrations were determined using the D_c_ protein assay kit (Bio-Rad), and 7.5 µg of protein per sample was prepared in SDS loading buffer and separated by 7.5% SDS‒PAGE. Subsequently, proteins were transferred overnight to a nitrocellulose membrane (Pall Corporation, New York, NY), which was probed using the indicated primary antibodies (a mouse monoclonal antibody recognizing residues 22-230 of human IP_3_R3 (BD Transduction Laboratories, San Jose, CA, 1/1000 dilution) and rabbit monoclonal antibody recognizing Calnexin (Cell Signaling Technology, Danvers, MA, 1/2000 dilution)) and corresponding secondary antibodies (goat anti-mouse/rabbit, Invitrogen, 1/10,000 dilution). Membranes were imaged with an Odyssey infrared imaging system (LICOR Biosciences, Lincoln, NE). The resulting scans were exported to LICOR Image Studio, where the band intensity was calculated. In Excel, the hR3 band intensity values were normalized to the value of the calnexin loading control. Subsequently, these values were further normalized to the value of the corresponding endogenous hIP_3_R3. Averages and statistical analysis were performed in GraphPad Prism.

### Plasmid cloning

All plasmids used in this study were based on the primary sequence of human IP_3_R3 in a pcDNA3.1 plasmid backbone. Mutagenesis to introduce base changes identified in the reported patients was performed with the primers listed in Supplementary Table 7, which had been synthesized by Integrated DNA Technologies. Suitable sequences were amplified by PCR and subcloned into a pJet1.2/blunt plasmid using the CloneJET PCR Cloning kit (Thermo Fisher) according to the manufacturer’s instructions. Mutagenesis PCR was performed with outward-facing complementary primers introducing the desired base change and amplifying the full plasmid. DpnI digestion in Tango Buffer (both from Thermo Scientific) was performed overnight, and the resulting circularized plasmids were used to transform chemically competent DH5α bacteria. Following ampicillin selection, the plasmids were purified from liquid cultures of single colonies using the NucleoSpin Plasmid EasyPure kit (Macherey-Nagel), and base exchange was verified by Sanger sequencing (Eurofins). Restriction enzyme-based cloning was then performed to introduce the mutated sequences into the original pcDNA3.1 construct, and the sequences were reverified.

### Generation of HEK-3KO cell lines stably expressing IP_3_R3 mutants

HEK-3KO cells are HEK293 cells engineered with CRISPR/Cas9 editing to delete three endogenous IP_3_R isoforms [44], and cells expressing endogenous IP_3_R3 are HEK293 cells modified with CRISPR/Cas9 editing to express only endogenous IP_3_R3. HEK-3KO cells, cells expressing endogenous IP_3_R3, and HEK-3KO cells stably expressing exogenous IP_3_R constructs were grown at 37 °C with 5% CO_2_ in DMEM supplemented with 10% fetal bovine serum, 100 U/mL penicillin, and 100 µg/mL streptomycin. The cell lines were maintained by subculturing as necessary, and any selection required was carried out using G418 (VWR, Radnor, PA). HEK-3KO transfection was performed similar to the previously described methods [27, 44]. In brief, 1 × 10^6^ cells were pelleted, washed once with PBS, and resuspended in an in-house transfection reagent containing ATP-disodium salt, MgCl_2_, KH_2_PO_4_, NaHCO_3_, and glucose at pH 7.4. DNA (2–5 µg) was mixed with the resuspended cells and electroporated using Amaxa cell nucleofector program Q-001. Cells recovered in fresh DMEM supplemented as described above for 48 h prior to culturing in new 10-cm dishes containing DMEM supplemented with 1.5–2 mg/mL G418. Approximately 7 days after the start of the selection, individual colonies were selected by hand and transferred to 24-well plates containing fresh DMEM with G418. Ten to fourteen days after transfection, wells that exhibited growth were expanded and screened by western blotting to verify the stable expression of the desired protein.

### Immunocytochemistry and confocal microscopy

HEK-3KO cells stably expressing exogenous IP_3_R constructs were plated on poly-d-lysine-coated coverslips. When approximately 50% confluent, the cells were fixed using 4% PFA at room temperature for 15 min. Subsequently, the coverslips were washed with PBS, and the cells were blocked in 10% bovine serum albumin (BSA) in PBS-T (PBS with 0.2% Tween 20) for 1.5 h. Following blocking, the cells were incubated with an anti-IP_3_R3 primary antibody in BSA overnight at 4 °C. The following day, the primary antibody was removed, and the coverslips were washed three times with PBS-T for 10 min with gentle rocking. Subsequently, a secondary antibody conjugated to Alexa Fluor 488 was incubated for 1 h in BSA at RT with gentle rocking. After incubation, the coverslips were washed with PBS-T and mounted on slides. After the slides were allowed to dry, the coverslips were sealed onto slides and imaged by confocal microscopy.

### Fluorescence measurement of calcium flux

Fibroblasts were seeded in 96-well plates (Greiner, Kremsmünster, Austria) and assessed for [Ca^2+^]_i_ using Fura2/AM (Eurogentec, Seraing, Belgium). Briefly, cells were loaded with the ratiometric fluorescent Ca^2+^ indicator Fura2/AM (1 µM) at RT for 30 min in a modified Krebs solution (containing 150 mM NaCl, 5.9 mM KCl, 1.2 mM MgCl_2_, 11.6 mM HEPES (pH 7.3), and 1.5 mM CaCl2). Cells were allowed to rest for 30 min at RT in the absence of Fura2/AM to allow complete dye de-esterification before analysis was performed with a FlexStation 3 microplate reader (Molecular Devices, Sunnyvale, CA, USA). The indicator was alternately excited at 340 and 380 nm, and fluorescence emission at 510 nm was recorded. EGTA was added after 30 s in all conditions to a final concentration of 3 mM to chelate Ca^2+^ ions in the buffer, and the fluorescence was recorded for 60 s prior to stimulation. The responses to stimuli prepared in Ca^2+^-free modified Krebs solution containing 3 mM EGTA were acquired for 6 min. Ionomycin and the irreversible sarco-/endoplasmic reticulum Ca^2+^-ATPase (SERCA) inhibitor thapsigargin were added to final concentrations of 10 µM, and bradykinin was added to a final concentration of 50 nM. All traces are shown as the ratio of both emission wavelengths F_340_/F_380_.

PBMCs from different donors were first stained with different anti-CD4 antibodies for multiplexing (all RPA-T4, eBioscience, BD Biosciences, Invitrogen). After mixing together, the PBMCs were loaded with ratiometric fluorescent Ca^2+^ indicator dye FuraRed AM (Thermo Fisher) as described above. For TCR stimulation experiments, the cells were incubated on ice with 10 µg/mL anti-CD3 (OKT3) and anti-CD28 antibodies (CD28.2, both eBioscience) for 30 min. Ionomycin and the irreversible SERCA inhibitor thapsigargin were each added to a final concentration of 10 µM, and a cross-linking goat anti-mouse IgG antibody (Abcam, Cambridge, UK) was added for TCR stimulation to a final concentration of 10 µg/mL.

The cells were analyzed on a BD FACSCanto flow cytometer (BD Biosciences, Franklin Lakes, NJ, US). The data on the ratio of emission at 450/50 nm after excitation at 405 nm and emission at 670 nm after excitation at 488 nm were exported using the Kinetics platform of FlowJo^TM^ software (v10.7.1, Ashland, OR). A gating strategy was used to select living single lymphocytes before distinguishing cells from different individuals based on the fluorochromes of the anti-CD4 antibodies, for details, please refer to Supplementary Fig. 1A. Raw data from both approaches were smoothened using a second-order running average of 5 in GraphPad Prism (v9.0.0, San Diego, CA). A baseline value was calculated for each measurement as the mean fluorescence during the 60 s before the addition of the stimulus. This baseline was used for calculating the area under the curve (AUC) and the peak amplitude using GraphPad Prism; the minimum peak height was 10% of the distance from the minimum to the maximum Y.

HEK-3KO cells and HEK-3KO cells stably expressing exogenous IP_3_R constructs were analyzed with a FlexStation 3 microplate reader (Molecular Devices) after loading with 4 µM Fura2/AM (TEFLabs, Austin, TX) in complete DMEM. After 1 h, the cells were harvested, washed, and resuspended in Ca^2+^ imaging buffer (10 mM HEPES; 1.26 mM Ca^2+^; 137 mM NaCl; 4.7 mM KCl; 5.5 mM glucose; 1 mM Na_2_HPO_4_; and 0.56 mM MgCl_2_, pH 7.4) before being dispensed into a black-walled flat-bottom 96-well plate (Greiner). The plate was centrifuged (200× g for 2 min) and incubated at 37 °C for 30 min prior to commencing the assay. Excitation was applied as described above, and Ca^2+^ imaging buffer or varying concentrations of CCh were added to induce IP_3_R-mediated Ca^2+^ release. Readings were taken every 4 s for a total of 200 s, and the data were exported from SoftMax® Pro Microplate Data Acquisition and Analysis software to Excel, where the F_340_/F_380_ ratio was calculated. The ratios were normalized to the average of the first five ratio values, and the AUC and peak amplitude were calculated in GraphPad Prism as described above. The data averages were derived on the basis of at least three individual plates, and the fit of each logistic curve was calculated with GraphPad Prism.

### NFAT translocation assay

Frozen PBMCs were thawed in RPMI 1640 (Gibco^TM^, Thermo Fisher) supplemented with 10% FBS (Tico Europe, Amstelveen, Netherlands) and 100 U/mL penicillin/streptomycin (Gibco^TM^, Thermo Fisher) and allowed to rest at 37 °C prior to incubation with 10 µg/mL anti-CD3 (OKT3) and 10 µg/mL anti-CD28 antibodies (CD28.2, both eBioscience) on ice for 30 min. Cross-linking goat anti-mouse IgG (Abcam) was added to a final concentration of 20 µg/mL for 30 min at 37 °C. As positive controls, 1 µM ionomycin and 50 ng/mL phorbol 12-myristate 13-acetate (PMA) were added and incubated for 30 min at 37 °C. Cells were fixed in 2% paraformaldehyde and permeabilized with 100% methanol prior to staining for CD4 (RPA-T4, eBioscience), CD8 (OKT8, Invitrogen), and NFAT1 (D43B1, Cell Signaling Technology) followed by AF488-conjugated secondary anti-rabbit IgG (Invitrogen, Thermo Fisher) and DAPI staining. Cells were acquired on an ImageStream^X^ MkII (Amnis Corporation) at 40x magnification. The data were analyzed using IDEAS software (Amnis Corporation), which enabled the identification of focused cells before gating on single DAPI^+^NFAT^+^CD4^+^ cells (see Supplementary Fig. 2). The percentage of cells with nuclear NFAT and the extent of NFAT translocation were analyzed with the Nuclear Localization Wizard, and the latter is presented as Similarity Score.

### Phosphorylation assay

Frozen PBMCs were thawed, plated, and rested in complete RPMI at 37 °C prior to stimulation with 5 µg/mL anti-CD3 (UCHT1) and 5 µg/mL anti-CD28 (CD28.2) antibodies (both from eBioscience) or anti-human IgA/IgG/IgM (Jackson ImmunoResearch, West Grove, PA) for the indicated durations. Cells were fixed in 2% paraformaldehyde and permeabilized with 100% methanol prior to staining for CD14 (M5E5, BioLegend), CD4 (RPA-T4, eBioscience), CD8 (SK1, eBioscience), CD19 (HIB19, eBioscience), and phospho-Erk (Thr202/Tyr204, MILAN8R, eBioscience). Cells were acquired on a BD FACSCanto flow cytometer (BD Biosciences). After exclusion of doublets and monocytes via CD14, T cells were gated as CD19^-^, and B cells were gated as CD4^-^CD8^-^CD19^+^ cells (Supplementary Fig. 1B). The fold change in the median fluorescence intensity was calculated based on the whole unstimulated population of the respective sample.

### Proliferation assay

Frozen PBMCs were thawed in RPMI 1640 (Gibco^TM^, Thermo Fisher) supplemented with 10% FBS (Tico Europe) and 100 U/mL penicillin/streptomycin (Gibco^TM^, Thermo Fisher) prior to labeling with 1 µM CFSE (Invitrogen^TM^, Carlsbad, CA) at 37 °C with regular shaking. The reaction was quenched with ice-cold FBS, and the cells were plated after washing in complete medium containing 10% FBS, 100 U/mL penicillin, 25 mM HEPES, and nonessential amino acids. T-cell stimulation was either performed in wells coated with 10 µg/mL anti-human CD3 (UCHT1) monoclonal antibody and 5 µg/mL anti-human CD28 (CD28.2) monoclonal antibody (both from eBioscience^TM^, San Diego, CA) supplemented in the medium or with both monoclonal antibodies, each at 5 µg/mL, supplemented in the medium. B-cell stimulation was performed with 10 µg/mL AffiniPure goat anti-IgA/IgG/IgM (Jackson ImmunoResearch) and 1 µg/mL CD40L with or without 20 ng/mL IL-21 (both from BioLegend). Cells were stained for CD4 (RPA-T4, Invitrogen), CD8 (OKT8, Invitrogen), and CD19 (HIB19, eBioscience) and analyzed by flow cytometry on a BD FACSCanto (BD Biosciences, Franklin Lakes, NJ, US) on days 3 and 4 after stimulation. For the gating strategy, refer to Supplementary Fig. 1C.

### Statistical analysis

All statistical analyses were done by performing ordinary one-way ANOVA with Dunnett’s multiple comparison test by comparing each individual to the healthy donors using GraphPad Prism 9.0.0 (San Diego, CA).

## Results

### Identification of compound heterozygous *ITPR3* variants in two immunodeficient patients

**Fig. 1:**
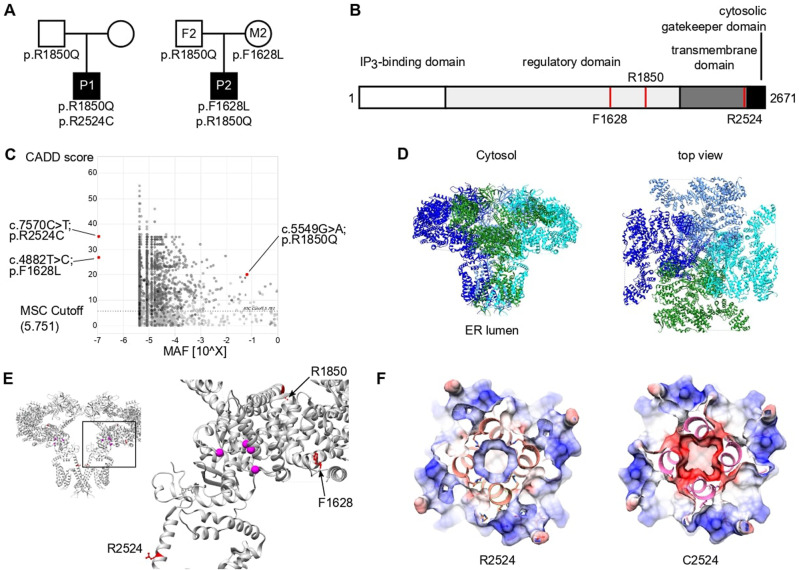
Identification of compound heterozygous variants in *ITPR3* in two patients with immunodeficiency or immune dysregulation. **A** Pedigrees of two unrelated families illustrating the genotype of the tested individuals. **B** Schematic domain structure of IP_3_R3 with positions of the variants indicated by red lines. **C** PopViz plot showing the mean allelic frequency (MAF) and the Combined Annotation Dependent Deletion (CADD) score with the identified variants in red. The dashed line indicates the gene-specific mutation significance cutoff (MSC). **D** Cryo-EM structure of homotetrameric IP_3_R3 (6DQJ) depicted in a ribbon diagram with different subunits in different colors. Two orthogonal views are shown: a side view along the membrane plane (left) and a view from the cytosol (right). **E** Illustration of the identified variants mapped onto the 3D structure of IP_3_R3 (6DQJ). Two opposing subunits are shown in side view. Unresolved sequences are indicated by dashed lines. The red dashed line spans a known phosphorylation region including the R1850 residue. Putative residues involving Ca^2+^ binding to the receptor are rendered as pink spheres. **F** Slices through the space-filled model of the IP_3_R3 TM region perpendicular to the 4-fold axis as seen from the cytosol. Surfaces are color-coded according to electrostatic charges calculated for the model (red: negative; blue: positive)

To identify the cause of immunodeficiency in two patients without a genetic diagnosis, comprehensive clinical, immunological, and genetic analyses were performed. Both patients were born to nonconsanguineous parents of European descent (Fig. 1A). P1 was a 12-year-old male patient who presented with combined immunodeficiency with profoundly low numbers of B and T cells and required hematopoietic stem cell transplantation (HSCT) at the age of 6 years. He developed an EBV-induced leiomyoma after transplantation and showed abnormal tooth eruption and mineralization as well as thin hair since birth. At the age of 11, P1 presented with peripheral neuropathy and was diagnosed with Charcot–Marie–Tooth. P2 was a 36-year-old male who presented with recurring immune thrombocytopenia (ITP), requiring splenectomy at the age of 19 years. He subsequently suffered from autoimmune hemolytic anemia, susceptibility to infections, and enteropathy. Hypogammaglobulinemia and low numbers of switched memory B cells led to a diagnosis of CVID and monthly treatment with intravenous immunoglobulin. The patient did not show signs of neuromuscular disorder. Additional clinical details are available in the Supplemental Data (extended clinical case descriptions, Supplementary Tables 1–3). None of the parents showed a clinically apparent immunological phenotype, and all self-reported to be healthy.

Whole-exome sequencing was performed for both patients, filtering for rare damaging variants after excluding the presence of known variants of inborn errors of immunity. We identified compound heterozygous candidate variants in *ITPR3* encoding inositol 1,4,5-trisphosphate receptor subtype 3 (IP_3_R3; NM_002224.4). Three distinct variants were identified in the two compound heterozygous patients. P1 harbored a de novo variant not reported in any public database but recently suggested to be a cause of Charcot–Marie–Tooth syndrome [31] (c.7570C>T:p.Arg2524Cys, referred to as R2524C). In addition, P1 inherited a c.5549G>A:p.Arg1850Gln substitution from his father that was also found in P2 and his father F2; this mutation is reported with an allelic frequency of 6%. P2 inherited a second private variant from his mother M2, resulting in a c.4882T>C:p.Phe1628Leu (referred to as F1628L) substitution. All variants were validated by Sanger sequencing (Supplementary Fig. 3A), and their position within the protein is depicted in Fig. 1B. All protein-coding variants were predicted to be damaging by Sorting Intolerant from Tolerant [45] and Deleterious Annotation of Genetic Variants Using Neural Networks [46] algorithms (Supplementary Table 4). In addition, all variants had a CADD score above the mutation significance cutoff (MSC 5.7) and were therefore predicted to exert a high impact on protein structure and function [47] (Fig. 1C). Importantly, there were no additional variants identified in the genes causing CRAC channelopathies, such as *ORAI1* or *STIM1*. These results demonstrate the identification of two reportedly unrelated patients with immunodeficiency and compound heterozygosity of predicted damaging *ITPR3* variants.

To further investigate the potential impact of the identified variants, we mapped them onto the IP_3_R3 structure resolved via cryo-EM [48, 49]. All the altered residues are highly conserved across species. Moreover, residues F1628 and R2524 are conserved among all three IP_3_R isoforms: IP_3_R1, IP_3_R2, and IP_3_R3 (Supplementary Fig. 3B, C). In vivo, these isoforms assemble as homo- or heterotetramers to form functional IP_3_-regulated Ca^2+^ channels in the ER membrane. Each IP_3_R monomer consists of a cytosolic N-terminal IP_3_-binding domain followed by a large coupling/regulatory multidomain region, a pore-forming region comprising six TM helices, and a C-terminal cytosolic tail [35, 48] (Fig. 1B, D). The F1628L and R1850Q variants are located in one of the regulatory alpha-helical domains. The R1850Q variant is close to the functionally important S1832 phosphorylation site [50], while F1628L is in close proximity to several predicted phosphorylation and ubiquitination sites [51] (Fig. 1E). The de novo R2524C variant is located in the sixth TM domain helix (S6). The S6 helices from each subunit shape the channel’s ion conduction pathway and form the constriction gate that controls ion translocation across the ER membrane. R2524 is located in the cytosolic extension of the S6 helix just beyond the gate [49], and its positive charge is likely neutralized through coordination with neighboring aspartate residues [52]. According to computational modeling, replacement of the positively charged arginine residue with an uncharged cysteine residue abolishes the charge coordination in the TM helix S6, thereby changing the pore charge in this region (Fig. 1F and Supplementary Fig. 3D). Due to the strong conservation of all the identified variants and their location in or close to predicted functionally relevant sites, we hypothesized that these variants alter channel function, leading to defective calcium mobilization and subsequent impaired lymphocyte activation.

### Functional impact of the *ITPR3* variants

**Fig. 2:**
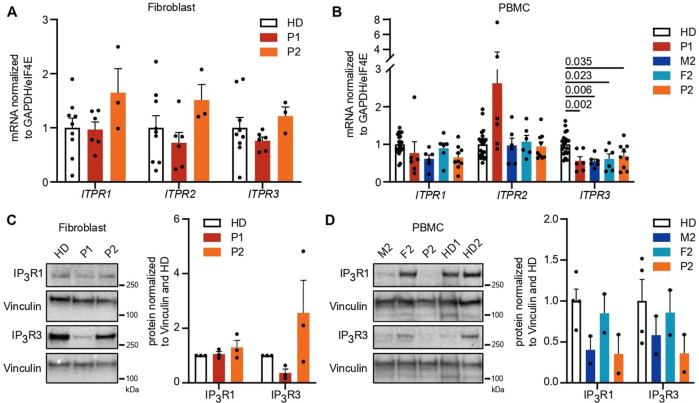
IP_3_R3 expression is reduced by variants in a cell type-dependent and variant-specific manner. **A**, **B** mRNA expression of all three receptor subtypes was measured in primary fibroblasts and PBMCs with isoform-specific primers. Dots represent the mean of duplicates for independent runs normalized to the mean values of the housekeeping genes GAPDH and eIF4E. Expression in **A** fibroblasts and **B** PBMCs. **C**, **D** Western blot analysis of IP_3_R1 (314 kDa) and IP_3_R3 (304 kDa) isoform protein expression was performed using subtype-specific antibodies. Representative blots with the molecular weight marker (left) indicated and the quantification of protein expression normalized to the housekeeping gene vinculin (124 kDa, right) are shown. Quantification was performed in **C** fibroblasts and **D** PBMCs. Number of independent experiments: **A** *n* = 3 with 2 different HDs. **B** *n* = 3 with 5 different HDs. **C** *n* = 3 with 2 different HDs. **D** *n* = 2 with 3 different HDs. Individuals were sampled at multiple time points. The values are presented as the mean + SEM. One-way ANOVA was performed with a multiple comparison test

To assess the functional impact of *ITRP3* variants, we investigated expression levels and calcium flux in patient fibroblasts and PBMCs. While PBMCs are more relevant to the observed phenotype in the probands, PBMCs were not available due to HSCT in P1. We therefore included fibroblasts to enable us to study primary cells from this patient, with the added advantage that measurements of intracellular Ca^2+^ fluxes are very sensitive and standardized by using automated pipetting devices with adherent cells such as fibroblasts. We first analyzed the influence of the variants on mRNA and protein expression levels of all IP_3_R subtypes in both cell types using isoform-specific primer pairs and antibodies selectively recognizing one of the isoforms or all of them simultaneously (pan-IP_3_R). mRNA expression was normal in fibroblasts (Fig. 2A); however, *ITPR3* levels were reduced in PBMCs from both patients and the parents of P2 (Fig. 2B). Immunoblot analysis yielded clear immunoreactive bands for IP_3_R1 and IP_3_R3 (Fig. 2C, D), while the signals for IP_3_R2 and an antibody recognizing all IP_3_R subtypes were weaker (Supplementary Fig. 4). Despite weak bands in some individual samples, expression levels were effectively quantified, as they were consistent across different experiments. In contrast to the mRNA levels, we detected reduced levels of IP_3_R3 in P1 fibroblasts (PBMCs were not available due to HSCT) (Fig. 2C). Protein expression levels in PBMCs were also variable among healthy donors, with a tendency toward reduced IP_3_R1 and IP_3_R3 in M2 and P2, consistent with the mRNA levels (Fig. 2D). While the expression of the IP_3_R subtypes and the impact of variants varied across investigated cell types, these results suggest a reduction of *ITPR3* in both patients at the mRNA and protein levels and in PBMCs of P2. The reduction of IP_3_R1 and IP_3_R3 levels in M2 and P2 may result from the F1628L variant alone and be a result of a baseline feedback mechanism acting at the transcriptional level, as mRNA levels were also reduced in both of these individuals.

**Fig. 3:**
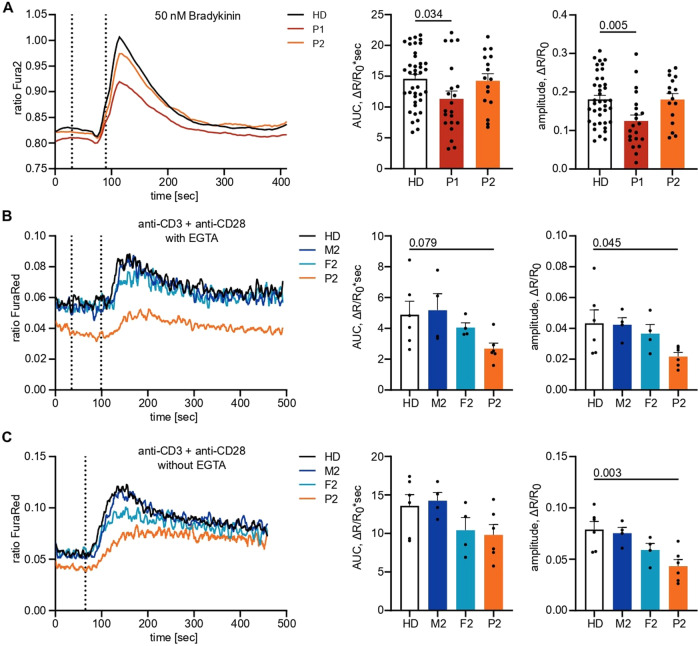
Variants in *ITPR3* impede IP_3_-mediated calcium signaling. **A** Adherent fibroblasts were loaded with the ratiometric Ca^2+^ indicator Fura2/AM, and calcium flux was monitored over time. The first dashed line indicates the addition of EGTA for the acquisition of the background, and the second dashed line indicates the addition of 10 µM bradykinin. The area under the curve (AUC) and maximal amplitude of the response were calculated with respect to individual background values. **B** PBMCs were stained for CD4, loaded with the ratiometric Ca^2+^ indicator FuraRed and preincubated with anti-CD3 and anti-CD28 on ice before monitoring changes in cytosolic Ca^2+^ concentrations at room temperature by flow cytometry. The first dashed line indicates the addition of EGTA, and the second dashed line indicates the addition of 10 µg/mL cross-linking antibody to induce TCR ligation in the absence of extracellular Ca^2+^. **C** The experiment was performed as in (**B**) with the first dashed line indicating the addition of 10 µg/mL cross-linking antibody to induce TCR ligation in the presence of extracellular Ca^2+^. Number of independent experiments: **A** *n* = 9 with 2 different HDs. **B**, **C** *n* = 3 with 3 different HDs. Individuals in **B**, **C** were sampled at multiple time points. Traces represent the mean values of all experiments, and response quantification is shown as the mean + SEM. One-way ANOVA was performed with a multiple comparison test

As IP_3_Rs are crucial for calcium dynamics and mediating IP_3_-induced Ca^2+^ flux, we sought to investigate Ca^2+^ responses in primary patient cells to test IP_3_R functionality. To this end, we first studied Ca^2+^ flux in patient fibroblasts cultured from skin biopsies and monitored the change in [Ca^2+^]_cyt_ in response to various stimuli using a ratiometric Ca^2+^ indicator (Fura2). Stimulation with the Ca^2+^ ionophore ionomycin and the sarco-/endoplasmic reticulum Ca^2+^-ATPase (SERCA) inhibitor thapsigargin in the presence of extracellular EGTA, a Ca^2+^-chelating agent, revealed dysregulated intracellular Ca^2+^ homeostasis in fibroblasts of P1 (Supplementary Fig. 5A, B). When directly monitoring IP_3_R-mediated Ca^2+^ release in fibroblasts in response to the physiological G-protein coupled receptor (GPCR) agonist bradykinin, we also observed a decreased response in P1, as assessed by the integrated response (area under the curve, AUC) as well as the peak amplitude normalized to the respective sample background (Fig. 3A). P2 displayed a comparable response to the HDs.

We then studied PBMCs to confirm impaired Ca^2+^ signaling by analyzing CD4^+^ T cells from P2 and his parents (P1 PBMCs were not available due to successful HSCT). By labeling CD4^+^ T cells from different donors with different fluorochromes before combining the samples, we minimized sample-to-sample variation. The cells were loaded with the ratiometric Ca^2+^ indicator FuraRed and stimulated with ionomycin or thapsigargin to investigate calcium homeostasis or anti-CD3 and anti-CD28 to assess physiological IP_3_R-mediated calcium responses. While Ca^2+^ homeostasis was disrupted in P2 and his parents (Supplementary Fig. 5C, D), physiological Ca^2+^ responses after TCR engagement were impaired only in P2, with F2 showing an intermediate phenotype (Fig. 3B, C). This finding illustrates insufficient subtype redundancy in lymphocytes to rescue the phenotype in P2, in contrast to the sufficient redundancy that was observed in fibroblasts. Together, these results suggest disrupted Ca^2+^ homeostasis and defects in IP_3_R-mediated Ca^2+^ release in both patients, with P1 potentially the more severe (also being detected in fibroblasts), although the direct comparison between patient PBMCs was not possible due to HSCT. Ca^2+^ homeostasis but not signaling defects were also observed in the heterozygous parents of P2.

**Fig. 4:**
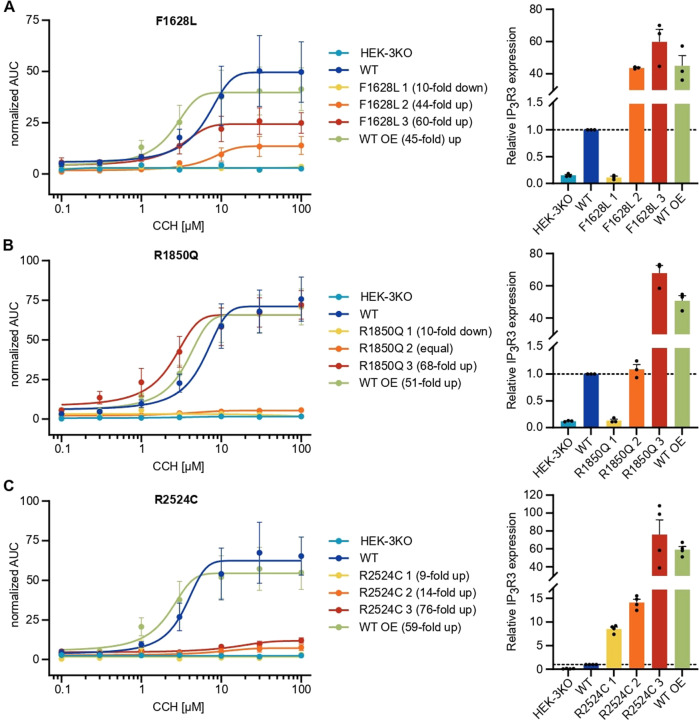
IP_3_-induced Ca^2+^ flux is reduced through F1628L-, R1850Q-, and R2524C-mutant IP_3_R3. HEK-3KO cells, not expressing any IP_3_R subtype, were stably transduced with the wild-type (WT OE – overexpressing) or mutated versions of the IP_3_R3 receptor, subcloned, and stimulated with different concentrations of the GPCR agonist carbachol (CCH) in Ca^2+^ imaging buffer. Cells expressing only endogenous IP_3_R3 (WT) and HEK-3KO cells were used as positive and negative controls, respectively. Dose–response curves of the AUC of representative cell lines stably expressing IP_3_R3 with the **A** F1628L (*n* = 3), **B** R1850Q (*n* = 3), or **C** R2524C (*n* = 3) variant are shown on the left. Fold changes in IP_3_R3 protein expression in transduced cells normalized to endogenous IP_3_R3 protein expression were determined after WB (for representative blots, see Supplementary Fig. 6B) and are indicated in brackets and plotted on the right. Dotted lines indicate the reference protein expression of the WT. Values are presented as the mean + SEM

To formally evaluate the impact of the *ITPR3* variants on calcium signaling in a model allowing direct comparison in the absence of compensatory mechanisms, we used HEK cells that had been engineered to eliminate endogenous expression of all three IP_3_R subtypes [44] (HEK-3KO) and reintroduced mutant IP_3_R3. Double-knockout of IP_3_R1 and IP_3_R2, resulting in a cell line expressing only endogenous IP_3_R3 (WT), was used as a control with physiological expression levels. WT IP_3_R3 in HEK-3KO cells was overexpressed (WT OE) to measure the maximum Ca^2+^ signaling capacity. We generated multiple monoclonal cell lines with transgenic expression of each mutant allele, confirmed the localization of each encoded protein to the ER via immunohistochemistry, and used western blotting to benchmark protein expression levels against endogenous WT expression (Supplementary Fig. 6A, B). Using these knockout cell lines reconstituted with mutant alleles, we investigated the calcium flux induced by different concentrations of the muscarinic GPCR agonist carbachol (CCH) and analyzed the magnitude of the responses (Supplementary Fig. 6C–E). The private F1628L variant of P2 inherited from his mother resulted in highly reduced responses even when protein expression was increased 40- to 60-fold compared to that of the endogenous WT (Fig. 4A and Supplementary Fig. 6F). Although the R1850Q variant shared by P1 and P2 as well as their fathers resulted in absent responses with protein levels similar to those of the WT, overexpression of more than 60-fold restored full channel function (Fig. 4B and Supplementary Fig. 6G). This outcome suggests that neither variant showed complete loss of function but instead display reduced signaling capacity. In contrast, the de novo R2524C variant identified in P1 showed more severe defects with absent Ca^2+^ mobilization across all the clones, suggesting complete loss of function (Fig. 4C and Supplementary Fig. 6H). Together, these results clearly demonstrate IP_3_R-mediated Ca^2+^ release defects in the F1628L, R1850Q, and R2524C mutants in an overexpression model, with the most striking defect found in the variant borne by P1, who presented with the more severe clinical manifestations of immunodeficiency.

### Defective immunological responses in *ITPR3* patients

**Fig. 5:**
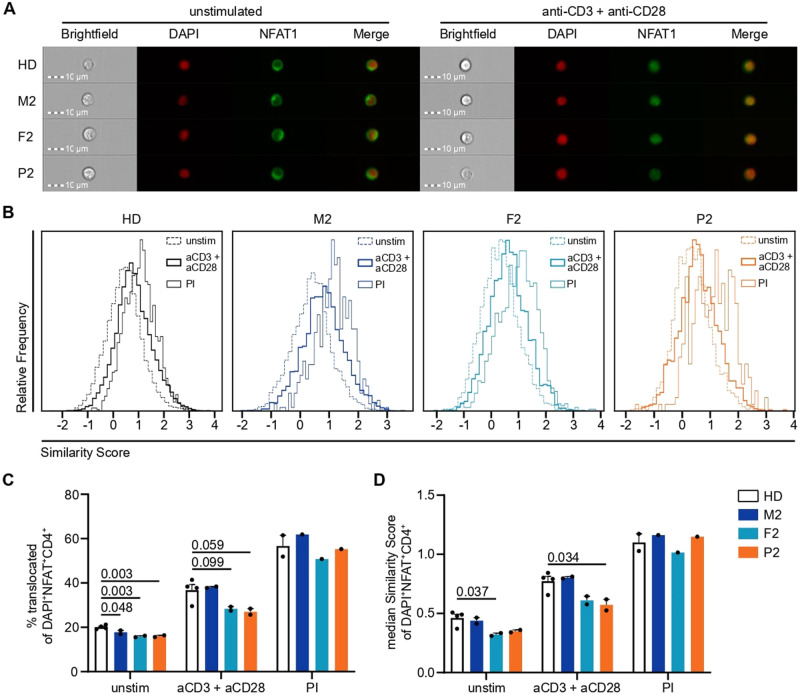
Nuclear translocation of NFAT1 is impaired by variants in IP_3_R3 following TCR but not PMA + ionomycin stimulation. PBMCs were incubated with anti-CD3 and anti-CD28 (both 10 µg/mL) antibodies on ice before cross-linking was induced for 30 min at 37 °C by adding 20 µg/mL of a cross-linking antibody for TCR stimulation (aCD3 + aCD28). PMA + ionomycin (PI) stimulation was performed simultaneously as a positive control. Nuclear translocation was assessed by ImageStream analysis in CD4^+^ T cells. **A** Representative images of unstimulated and TCR-stimulated cells from all the individuals. **B** Histograms of the Similarity Score as a measure of nuclear NFAT1 localization for unstimulated, TCR-stimulated, and PI-stimulated CD4^+^ T cells. **C** Percentage of unstimulated, TCR-stimulated, and PI-stimulated CD4^+^ T cells with nuclear NFAT1. **D** Median Similarity Score of unstimulated, TCR-stimulated, and PI-stimulated CD4^+^ T cells. Number of independent experiments: *n* = 2 with 4 different HDs for unstimulated and TCR-stimulated cells, *n* = 1 with 2 different HDs for PI-stimulated cells. Individuals were sampled at different time points. The values are presented as the mean + SEM. One-way ANOVA was performed with a multiple comparison test

Following our observations that Ca^2+^ signaling was disrupted in immune cells of our patients, we investigated the downstream effects on immune cell function. P1 was, of necessity, excluded due to successful transplantation of the hematopoietic compartment. First, we investigated the impact of variants in *ITPR3* on early downstream Ca^2+^-dependent translocation of NFAT from the cytoplasm to the nucleus, where it acts as a transcription factor to induce the expression of, among other genes, IL-2, which is crucial for a productive immune response [53, 54]. TCR engagement by cross-linking anti-CD3 and anti-CD28 antibodies resulted in translocation of NFAT1 to the nucleus (Fig. 5A). Quantification of the translocation response was performed by calculating the Similarity Score, which is a measure of correlated pixels between stained NFAT1 and the DAPI-stained nucleus. P2 showed impaired nuclear NFAT1 translocation compared to the healthy controls, but translocation after stimulation with PMA and ionomycin was normal (Fig. 5B–D). Although the mother responded normally, translocation in F2 was impaired, which was in line with the aforementioned intermediate phenotype in the Ca^2+^ response.

**Fig. 6:**
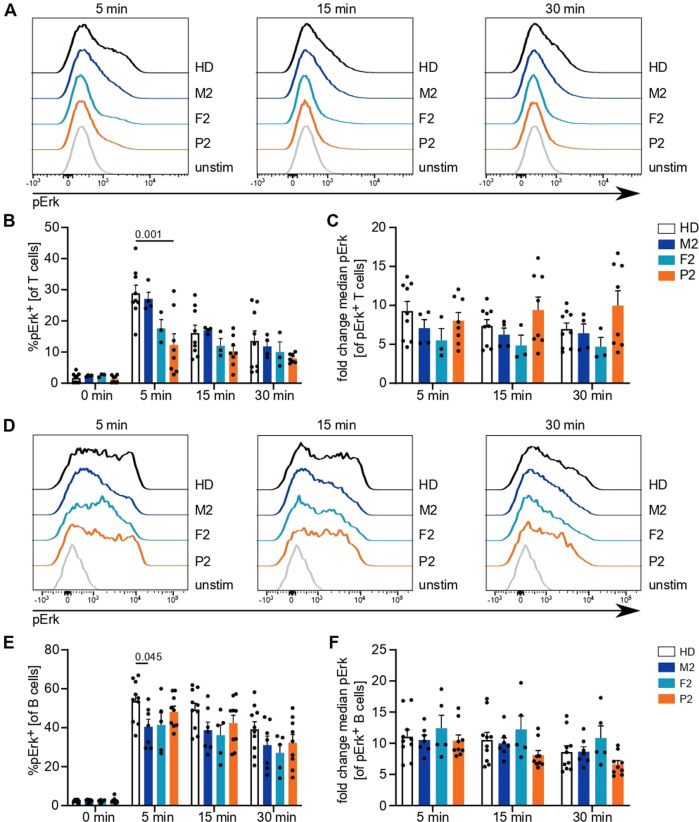
Antigen receptor downstream phosphorylation events are reduced in P2. PBMCs were stimulated with anti-CD3 and anti-CD28 antibodies or immunoglobulins for 5, 15, or 30 min. **A** Representative histograms illustrating Erk phosphorylation following TCR stimulation after different stimulation intervals. **B** Percentage of T cells with phosphorylated Erk among (un)stimulated cells. **C** Fold change of median fluorescence intensity in pErk^+^ cells compared to that in the total unstimulated cell population. **D** Representative histograms illustrating Erk phosphorylation following BCR stimulation after different stimulation intervals. **E** Percentage of B cells with phosphorylated Erk in (un)stimulated cells. **F** Fold change of median fluorescence intensity in pErk^+^ cells compared to that in the total unstimulated cell population. Number of independent experiments: **A**–**C** *n* = 4 with 4 different HDs. **D**–**F** *n* = 6 with 5 different HDs. Individuals were sampled at multiple time points. The values are presented as the mean + SEM. One-way ANOVA was performed with a multiple comparison test

Next, we investigated activation by phosphorylation of another important protein mediating immune cell activation, namely, Erk. Following TCR stimulation, the percentage of pErk^+^ positive cells was reduced in P2 at different time points, while the heterozygous parents presented an intermediate phenotype (Fig. 6A, B). Cells that responded showed comparable phosphorylation of Erk (Fig. 6C), indicating that the defect was in triggering pathway activation. Stimulation of the BCR resulted in relatively normal pErk positivity in B cells (Fig. 6D–F). These results suggest a functional defect of IP_3_R3 in P2 resulting in impaired TCR signaling.

**Fig. 7:**
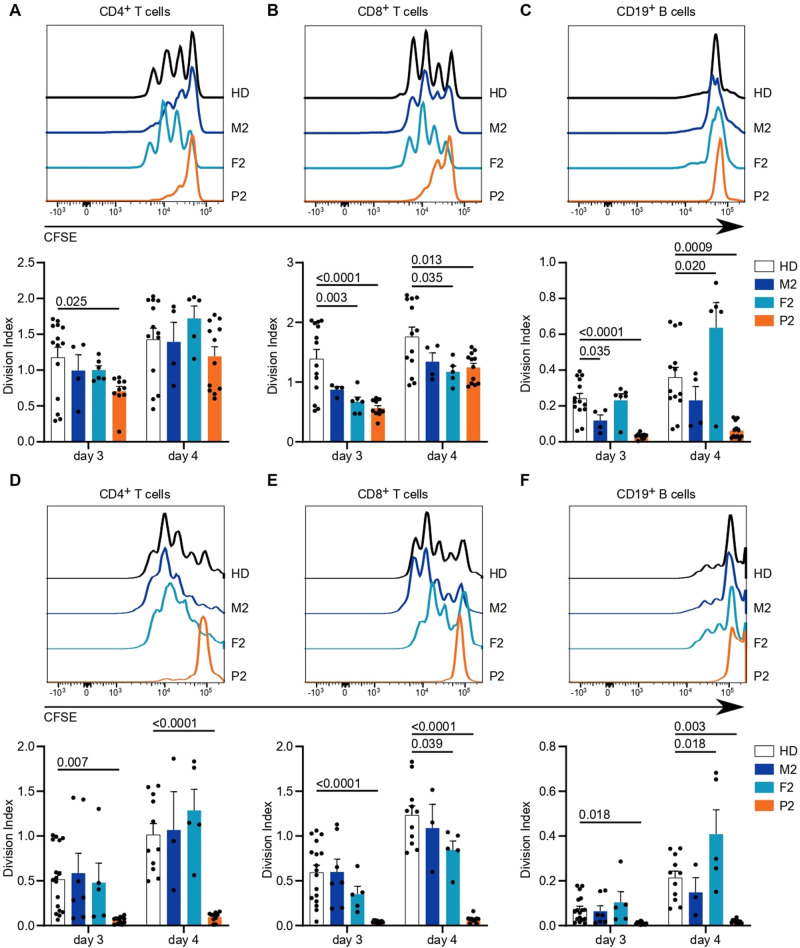
Altered cellular responses with *ITPR3* variants. PBMCs were labeled with the CFSE cell proliferation dye and stimulated with anti-CD3 and anti-CD28 antibodies for up to 4 days. The mean number of proliferations in the whole cell population, including undivided cells, was calculated for CD4^+^, CD8^+^, and CD19^+^ cell subsets (Division Index). Stimulation was performed in plates coated with 10 µg/mL anti-CD3 antibody and 5 µg/mL soluble anti-CD28 antibodies. Exemplary histograms are shown on top, and the Division Indexes are shown on the bottom for **A** CD4^+^ T cells, **B** CD8^+^ T cells, and **C** B cells, with the latter reading indicating bystander activation and not direct stimulation. **D** Stimulation was performed with 5 µg/mL soluble anti-CD3 and 5 µg/mL soluble anti-CD28 antibodies. Exemplary histograms (top) and Division Indexes (bottom) are shown for CD4^+^ T cells, **E** CD8^+^ T cells and **F** B cells. Number of independent experiments: **A**–**C** *n* = 4 with 3 different HDs. **D**–**F** *n* = 5 with 5 different HDs. Individuals were sampled at multiple time points. The values are presented as the mean + SEM. One-way ANOVA was performed with a multiple comparison test

Finally, we assessed the downstream results of lymphocyte activation by analyzing the proliferative potential of CD4^+^ and CD8^+^ T cells in response to TCR engagement and CD19^+^ B cells in response to BCR engagement. The total cell numbers after TCR stimulation were reduced in all individuals in the kindred of P2 compared to healthy donors (Supplementary Fig. 7A, B). TCR-induced cell division was substantially reduced in CD4^+^ and CD8^+^ T cells of P2, with intermediate phenotypes acquired by the heterozygous parents (Fig. 7A, B and Supplementary Fig. 7C, D). The reduced proliferative capacity of P2 T cells was even more pronounced when PBMCs were stimulated with soluble anti-CD3 and soluble anti-CD28 antibodies, mimicking suboptimal stimulation conditions (Fig. 7D, E and Supplementary Fig. 7F, G). Interestingly, bystander activation of CD19^+^ B cells [55] was also profoundly impeded in P2 cells (Fig. 7C, F and Supplementary Fig. 7E, H), which might result from reduced cytokine production by fewer and less activated T cells. Direct stimulation of B cells with anti-Ig and CD40L with or without IL-21 only showed a trend toward impairment in B-cell proliferation (Supplementary Fig. 7I, J). Taken together, our results demonstrate impaired immunological responses, especially to TCR stimulation, in P2, with weak effects observed in the heterozygous parents, consistent with the defects in calcium flux and phosphorylation of Erk.

## Discussion

**Fig. 8:**
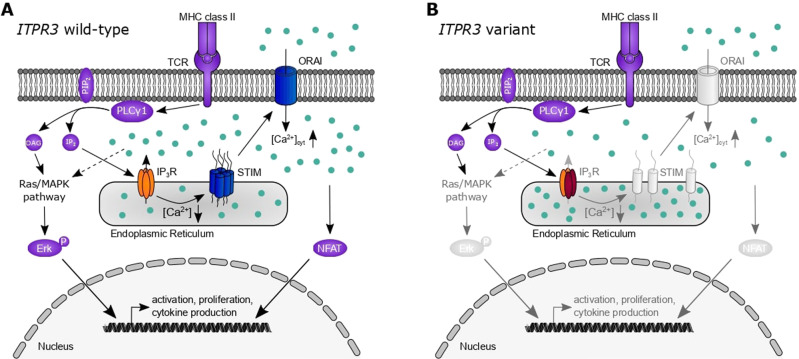
Dysfunctional IP_3_R3 subunits impair channel function and T-cell activation. Simplified schematic representation of T-cell activation pathways relevant to the presented results. **A** T-cell receptor (TCR) stimulation results in the activation of phospholipase C (PLC) γ and the subsequent generation of diacylglycerol (DAG) and inositol 1,4,5-trisphosphate (IP_3_) from phosphatidylinositol 4,5-bisphosphate (PIP_2_). While DAG activates the Ras/mitogen-activated protein kinase (MAPK) pathway and ultimately phosphorylation of Erk, allowing its nuclear translocation, IP_3_ binds to the tetrameric IP_3_ receptor (IP_3_R). Its activation results in Ca^2+^ (light blue dots) efflux from the endoplasmic reticulum, which in turn activates STIM and the opening of the plasmalemmal ORAI channels that mediate store-operated Ca^2+^ influx. Variants in both of these proteins (dark blue) have been previously described in the human immunodeficiency context. The influx from extracellular Ca^2+^ through ORAI results in an increase in cytosolic Ca^2+^ ([Ca^2+^]_cyt_) that results in nuclear translocation of nuclear factor of activated T cells (NFAT) and other effects. These processes result in the transcription of essential genes for cell activation, proliferation, and cytokine production. **B** Damaging variants of IP_3_R3 (dark red subunits) result in impaired function of homo- or heterotetrameric receptor complexes with reduced Ca^2+^ efflux into the cytosol. Reduced signaling via STIM/ORAI and impaired activation of Erk and NFAT pathways are insufficient for adequate lymphocyte activation and manifest in clinical immunodeficiency

Store-operated Ca^2+^ entry has been established to be a critical event in immune cell activation, with disruption resulting in human diseases described as CRAC channelopathies [56]. In this work, we identify variants in IP_3_R3, a channel mediating Ca^2+^ release from the ER into the cytosol prior to and necessary for store-operated Ca^2+^ entry initiation, resulting in similar clinical phenotypes that are characterized by combined immunodeficiency. Three different variants in two unrelated compound heterozygous patients demonstrated impaired IP_3_-mediated Ca^2+^ responses in vitro, translating into deficient T-cell activation and proliferation (Fig. 8). Our results suggest a more severe impact on channel function induced by the R2524C variant, which was consistent with the clinically more severe combined immunodeficiency observed in P1. For P2, who harbored the F1628L and R1850Q variants, we found defects in all in vitro assays, with partially reduced sensitivity of channel activation correlating with a weaker clinical phenotype. Although decreased protein expression of IP_3_R1 and IP_3_R3 in P2 was also observed in his mother and may therefore be an effect specifically attributed to the F1628L variant, this reduction was not sufficient to induce proliferation defects in M2. Similarly, although Ca^2+^ homeostasis was impaired in the clinically healthy heterozygous parents of P2 in a dominant way, IP_3_-mediated Ca^2+^ signaling and the integration of stimulating signals were intact in the parents. Together, these results suggest a spectrum of escalating clinical severity with progressive decline in channel function.

*ITPR3* belongs to an intriguing class of immunodeficiency genes involved in common cellular pathways. While defects in lymphocyte-specific processes, such as VDJ recombination, show a clear route to immunodeficiency, multiple immunodeficiencies have also been described based on genes in pathways such as DNA replication and cellular metabolism pathways, or shared signaling pathways such as Ca^2+^ signaling pathways [24]. Complete defects in these processes often manifest in embryonic lethality; therefore, the clinically relevant variants observed in these pathways must exist within a narrow band of mutational space that permits normal development but causes defects in cell types not necessary for development. The breadth of this mutational space varies by gene and influences the frequency at which clinically relevant variants arise. In the case of *ITPR3*, these constraints would require that variants exert a sufficient impact on a clinically relevant cell type, such as T cells and B cells, while maintaining sufficient functionality in developmental cell types, such as fibroblasts and neurons. This mutational balance could potentially be achieved via several different molecular mechanisms. The observation that the effect of a variant on mRNA expression differs between fibroblasts and T cells is consistent with early reports suggesting distinct subtype-specific expression profiles in various cell lines [57, 58]. Alternatively, the IP_3_R subtypes have been reported to exhibit differential regulatory and functional properties [26, 35, 59–61], which may account for the lack of a Ca^2+^ signaling phenotype in the fibroblasts of P2. The F1628L and R1850Q variants of P2 are located in the regulatory domain known for functioning as a signaling hub and interacting with a plethora of proteins [62, 63]. Considering that this domain is less conserved among subtypes, compared to, for example, the IP_3_-binding or TM domain [64], enhanced diversity of interaction partners may drive the acquisition of different phenotypes of mutated proteins, with a subset of these interactions disrupted. In this scenario, even variants in a single IP_3_R3 subunit in a heterotetramer may impair channel function in a specific cell type due to a reduced ability to bind a cooperative partner necessary for channel activation in that cell type [44, 65]. Further studies on the interaction partners required in different cell types may be aided by the identification of variants such as those presented herein, which display cell type-specific defects in signaling function.

We propose a partially recessive mode of inheritance for the F1628L (M2 and P2) and R1850Q (P1, F2, and P2) variants. This recessive inheritance may be explained via the location of the variants in the regulatory domain of IP_3_R3. Point mutations in this domain may be expected to impair only a specific aspect of regulation, which can be partially, but not entirely, compensated for, leading to impaired Ca^2+^ homeostasis in vitro even in heterozygous individuals under steady-state conditions. Despite the relatively high allelic frequency of the R1850Q variant, we present evidence that this mutant impairs channel function in a homotetramer without causing a clinical phenotype in heterozygous individuals due to partial compensation. While clinically silent, we postulate that the partial loss-of-function of one allele through R1850Q allows phenotypes to emerge when pathogenic variants constitute the alternative allele. This mode of inheritance is similar to that recently described for an adult patient with compound heterozygous variants of CRACR2A, a regulator of store-operated Ca^2+^ entry, who presented with T-cell dysfunction and hypogammaglobulinemia with normal in vitro B-cell function [66]. In this case, each of the variants was inherited from one clinically healthy parent, suggesting compensation of a heterozygous defect. In this model, the compound heterozygous loss of two regulatory mechanisms in IP_3_R3 was sufficient to induce combined immunodeficiency in P2. A similar inheritance pattern may account for the effect of the R2524C variant in P1; however, the de novo nature of the second variant precluded clear characterization of the R2524C variant as recessive. The R2524C allele has, however, been implicated with dominant inheritance for Charcot–Marie–Tooth, as reported in Rönkkö et al. [31]. The absence of a reported immunological phenotype in this patient, who inherited only a R2524C variant, suggests that a second hit with the potentiating R1850Q variant is necessary for immunodeficiency.

In addition to insights into the differential sensitivity of cell types to IP_3_R3 deficiency and the identification of the R1850Q, F1628L, and R2524C variants as pathogenic, this study provides potential insight into the molecular regulation of IP_3_R3. Despite advances in the accuracy of electron microscopy techniques that can reveal conformational changes and interactions in specific activation states of IP_3_Rs [48, 49, 52], receptor regulation remains incompletely understood, with the effects of posttranslational modifications and interactions with accessory proteins being continuously discovered [62]. Genetic variants leading to human disease provide a unique way for better understanding protein regulation and function. Our results demonstrate that the F1628L (P2) and R1850Q (P1 and P2) variants each result in reduced sensitivity to channel activation while still maintaining channel gating ability. In contrast, we see that the de novo R2524C variant in P1 appears to have a biochemically distinct effect, completely abolishing the gating of a homotetrameric receptor. This may therefore exert a dominant negative effect on channel function in heterotetramers. The conserved residue analogous to R2524C in the related Ryanodine receptor family has been shown to stabilize both the closed and open state of a channel by alternating its interaction with two other highly conserved residues [67] and has recently been identified as a crucial residue for IP_3_R3 channel activation in humans [68]. In line with the prediction of a drastic change in the pore charge introduced by this variant, its counterpart in the rat IP_3_R1 protein has been suggested to exhibit a crucial function in signal transmission, as mutation into different amino acids markedly reduced channel function [52, 69]. Further identification of pathogenic *ITPR3* variants and systematic investigation of these mutated receptors may reveal protein interactions and/or conformational changes that are hampered in different cell types, providing better insight into channel regulation.

With this study, we add variants in *ITPR3* as underlying causes for disrupted Ca^2+^ signaling that results in immunodeficiency. Some clinical characteristics of the patients we describe overlap with those of patients suffering from ORAI1 and STIM1 deficiency, who present with severely reduced or absent store-operated Ca^2+^ entry in vitro and normal lymphocyte counts but impaired T-cell proliferation following in vitro stimulation [2, 19]. Comparable to the patients we describe, ORAI1- and STIM1-deficient patients suffer from recurrent severe infections and can exhibit signs of lymphoproliferation and autoimmune disease, although the disease course is often more severe, with patients requiring HSCT very early in life to prevent a lethal disease course [70, 71]. Clinical presentation does, however, vary greatly in STIM1 deficiency, with several STIM1-deficient patients described with late-onset or absent clinical immunodeficiency [72, 73]. In P1, we observed nonimmunological disease manifestations, including dental mineralization defects, abnormalities of the hair and Charcot–Marie–Tooth disease. These nonimmunological effects, also observed in an unrelated R2524C patient, partially overlap with the dental enamel formation defects and myopathy in ORAI1 and STIM1 patients [19, 72, 74, 75], although the latter phenotypes are congenital and nonprogressive.

Here, we provide evidence showing that inherited variants in *ITPR3* alter physiologically relevant Ca^2+^ signaling responses, driving defects in immune responses, and are associated with clinical immunodeficiency. Limitations of our work include the limited number of cases, only two patients, and the lack of primary immune cells from P1 due to successful HSCT. Because of the inheritance pattern, we also lacked primary immune cells harboring only the R2524C variant, that would have allowed us to clarify the recessive or dominant nature of this mutation in lymphocytes. The identification of new patients in subsequent studies will be highly informative for identifying the mutational space in *ITPR3* associated with immunodeficiency. Additional studies investigating the effect of *ITPR3* variants on cellular processes such as apoptosis and autophagy, which are known to be regulated by IP_3_Rs coupling Ca^2+^ signaling between the ER and mitochondria, will also be interesting [76, 77]. Any defects in these pathways may alter immune cell activation and potentially contribute to the clinical presentations observed. While additional studies are needed, we propose that *ITPR3* should be considered as a candidate gene in patients with B- and T-cell defects in association with impaired Ca^2+^ signaling function, adding variants in *ITPR3* as causes of inborn errors of immunity.

## Supplementary information

Supplementary Material
