# Author Correction: Disrupted Ca^2+^ homeostasis and immunodeficiency in patients with functional IP_3_ receptor subtype 3 defects

**DOI:** 10.1038/s41423-022-00960-4

**Published:** 2022-12-06

**Authors:** Julika Neumann, Erika Van Nieuwenhove, Lara E. Terry, Frederik Staels, Taylor R. Knebel, Kirsten Welkenhuyzen, Kourosh Ahmadzadeh, Mariah R. Baker, Margaux Gerbaux, Mathijs Willemsen, John S. Barber, Irina I. Serysheva, Liesbeth De Waele, François Vermeulen, Susan Schlenner, Isabelle Meyts, David I. Yule, Geert Bultynck, Rik Schrijvers, Stephanie Humblet-Baron, Adrian Liston

**Affiliations:** 1 VIB Center for Brain and Disease Research, Leuven, Belgium; 2 Department of Microbiology and Immunology, KU Leuven, Leuven, Belgium; 3 UZ Leuven, Leuven, Belgium; 4 Department of Pharmacology and Physiology, University of Rochester, Rochester, NY 14526 USA; 5 Laboratory of Molecular and Cellular Signaling, Department of Cellular and Molecular Medicine, Leuven Kankerinstituut, KU Leuven, Leuven, Belgium; 6 Laboratory of Immunobiology, Department Microbiology and Immunology, Rega Institute, KU Leuven, Leuven, Belgium; 7 Department of Biochemistry and Molecular Biology, Structural Biology Imaging Center, McGovern Medical School at The University of Texas Health Science Center at Houston, Houston, TX 77030 USA; 8 Pediatric Department, Academic Children Hospital Queen Fabiola, Université Libre de Bruxelles, Brussels, Belgium; 9 Department of Pediatric Neurology, University Hospitals Leuven, Leuven, Belgium; 10 Department of Pulmonology, University Hospitals Leuven, Leuven, Belgium; 11 Laboratory for Inborn Errors of Immunity, Department of Immunology and Microbiology, KU Leuven, Leuven, Belgium; 12 Laboratory for Allergy and Clinical Immunology and Immunogenetics Research Group, Department of Microbiology, Immunology and Transplantation, KU Leuven, Leuven, Belgium; 13 Immunology Programme, The Babraham Institute, Babraham Research Campus, Cambridge, CB22 3AT UK

**Keywords:** Adaptive immunity, Primary immunodeficiency disorders, Calcium signalling, Immunogenetics

Correction to: *Cellular & Molecular Immunology* 10.1038/s41423-022-00928-4, published online 27 October 2022

In this article the statement in the Acknowledgements section was incorrectly given as “MRB and IIS are supported by the NIH R01GM080139 grant (to IIS), the Welch Foundation Research Grant AU-2014-20190331 (to IIS), and the American Heart Association grant 18CDA34110086 (to MRB)” and should have been, “MRB and IIS are supported by the NIH R01GM072804 grant (to IIS), the Welch Foundation Research Grant AU-2014-20190331 (to IIS), and the American Heart Association grant 18CDA34110086 (to MRB).”

The original article has been corrected.

